# *N*-[4-(4-Nitro­phen­oxy)phen­yl]propionamide

**DOI:** 10.1107/S1600536808034119

**Published:** 2008-10-25

**Authors:** Asifa Nigar, Zareen Akhter, Michael Bolte, Humaira M. Siddiqi, Rizwan Hussain

**Affiliations:** a Department of Chemistry, Quaid-I-Azam University, Islamabad 45320, Pakistan; b Institut für Anorganische Chemie, J.W. Goethe-Universität Frankfurt, Max-von-Laue-Strasse 7, 60438 Frankfurt/Main, Germany; c NESCOM, PO Box 2216, Islamabad, Pakistan

## Abstract

The title compound, C_15_H_14_N_2_O_4_, is an important inter­mediate for the synthesis of thermotropic liquid crystals. The dihedral angle between the two aromatic rings is 84.29 (4)°. An N—H⋯O hydrogen bond connects the mol­ecules into chains running along the *b* axis. In addition, the crystal packing is stabilized by weak C—H⋯O hydrogen bonds.

## Related literature

For background on liquid crystals, see: Bahadur (1992 ▶); Collings (1990 ▶); Collings & Hird (1997 ▶). For bond lengths and angles in organic compounds, see: Allen *et al.* (1995 ▶). For related literature, see: Akhter *et al.* (2007 ▶); Cârlescu *et al.* (2005 ▶).
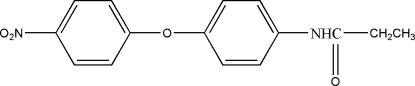


## Experimental

### 

#### Crystal data

C_15_H_14_N_2_O_4_
*M* *_r_* = 286.28
Monoclinic, 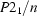

*a* = 14.8597 (14) Å
*b* = 5.2400 (3) Å
*c* = 17.9034 (16) Å
β = 101.875 (7)°
*V* = 1364.21 (19) Å^3^
*Z* = 4
Mo *K*α radiation
μ = 0.10 mm^−1^
*T* = 173 (2) K
0.37 × 0.28 × 0.19 mm

#### Data collection

Stoe IPDSII diffractometer
Absorption correction: none
16399 measured reflections
2788 independent reflections
2347 reflections with *I* > 2σ(*I*)
*R*_int_ = 0.048

#### Refinement

*R*[*F* ^2^ > 2σ(*F* ^2^)] = 0.036
*wR*(*F* ^2^) = 0.096
*S* = 1.03
2788 reflections
195 parameters
H atoms treated by a mixture of independent and constrained refinement
Δρ_max_ = 0.28 e Å^−3^
Δρ_min_ = −0.19 e Å^−3^

### 

Data collection: *X-AREA* (Stoe & Cie, 2001 ▶); cell refinement: *X-AREA*; data reduction: *X-RED* (Stoe & Cie, 2001 ▶); program(s) used to solve structure: *SHELXS97* (Sheldrick, 2008 ▶); program(s) used to refine structure: *SHELXL97* (Sheldrick, 2008 ▶); molecular graphics: *XP* in *SHELXTL-Plus* (Sheldrick, 2008 ▶); software used to prepare material for publication: *SHELXL97*.

## Supplementary Material

Crystal structure: contains datablocks I, global. DOI: 10.1107/S1600536808034119/wm2199sup1.cif

Structure factors: contains datablocks I. DOI: 10.1107/S1600536808034119/wm2199Isup2.hkl

Additional supplementary materials:  crystallographic information; 3D view; checkCIF report

## Figures and Tables

**Table 1:** Hydrogen-bond geometry (Å, °)

*D*—H⋯*A*	*D*—H	H⋯*A*	*D*⋯*A*	*D*—H⋯*A*
N1—H1⋯O1^i^	0.825 (17)	2.255 (17)	3.0306 (13)	156.7 (15)
C25—H25⋯O3^ii^	0.95	2.42	3.2082 (17)	140
C23—H23⋯O4^iii^	0.95	2.53	3.3400 (16)	144

Symmetry codes: (i) 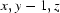; (ii) 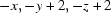; (iii) 
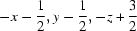
.

**Fig. 1.**
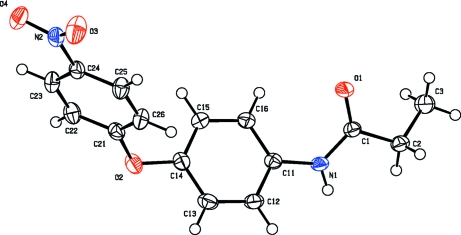
The molecule of the title compound with atom labelling and displacement ellipsoids drawn at the 50% probability level. H atoms are given as spheres of arbitrary radius.

## supplementary crystallographic information

### Comment

Liquid crystals are materials which exhibit phases where molecular order is intermediate between that of an ordered solid and a disordered liquid. They represent the combined properties of both the crystalline state (*e.g.* optical and electrical anisotropy) and the liquid state (*e.g.* molecular mobility and fluidity). The two major classes of liquid crystals are thermotropic and lyotropic, which can be distinguished by the mechanism that drive their self-organization. Background information on liquid crystals and their various applications were surveyed, for example, by Collings (1990), Bahadur (1992), and Collings & Hird (1997). One of the basic characteristics for the establishment of the mesophase is the ratio of rigid and flexible portions in the main structure (Cârlescu *et al.*, 2005). Therefore such type of precursors can be used to study structure property relationship of the liquid crystalline materials.

The crystal structure of the compound reported here is an important intermediate for the synthesis of thermotropic liquid crystals (Akhter *et al.*, 2007).

In the molecule of the title compound (Fig. 1), the bond lengths (Allen *et al.*, 1995) and angles are within normal ranges.

The dihedral angle between the two aromatic rings is 84.29 (4)°. An N—H···O hydrogen bond connects the molecules to chains running along the *b* axis. In addition, the crystal packing is stabilized by weak C—H···O hydrogen bonds.

### Experimental

A mixture of 5.046 g (50 mmol) 4-aminophenol, 6.91 g (50 mmol) anhydrous K_2_CO_3_ and 5.3 ml (50 mmol) 4-nitrofluorobenzene in 70 ml DMF was heated at 373 K for 18 h in an inert atmosphere. After cooling to room temperature, the reaction mixture was poured into 800 ml of water to yield a yellow solid. The product was filtered, dried and then re-crystallized from n-hexane (yield 86%). In a second step, propanoic acid and thionylchloride were refluxed in equimolar amounts for 30 min before evaporating excessive thionylchloride with a vacuum pump. The above prepared 4-[4-nitrophenoxy]aniline was then added to the propanoyl chloride solution in dry THF. 1 ml of triethylamine was also added for 1 g of 4-[4-nitrophenoxy]aniline and refluxed for 2 h under inert conditions. The reaction mixture was allowed to stand at room temperature overnight and filtered off the salt formed. The filterate was evaporated using a rotary evaporator, and the crude product obtained was re-crystallized from toluene (yield 76%, m.p. 416 K).

### Refinement

All H atoms could be located from difference Fourier maps. Except the amino H atom that was freely refined, all other H atoms were refined using a riding model with *U*_iso_(H) = 1.2*U*_eq_(C) and *U*_iso_(H) = 1.5*U*_eq_(C_methyl_) and distance restraints of C—H(aromatic) = 0.95 Å, C—H(methyl) = 0.98 Å and C—H(methylene) = 0.99 Å, respectively.

### Crystal data

**Table:**

C_15_H_14_N_2_O_4_	*F*(000) = 600
*M**_r_* = 286.28	*D*_x_ = 1.394 Mg m^−^^3^
Monoclinic, *P*2_1_/*n*	Mo *K*α radiation, λ = 0.71073 Å
Hall symbol: -P 2yn	Cell parameters from 14480 reflections
*a* = 14.8597 (14) Å	θ = 3.5–26.6°
*b* = 5.2400 (3) Å	µ = 0.10 mm^−^^1^
*c* = 17.9034 (16) Å	*T* = 173 K
β = 101.875 (7)°	Plate, yellow
*V* = 1364.21 (19) Å^3^	0.37 × 0.28 × 0.19 mm
*Z* = 4	

### Data collection

**Table:**

Stoe IPDSII diffractometer	2347 reflections with *I* > 2σ(*I*)
Radiation source: fine-focus sealed tube	*R*_int_ = 0.048
graphite	θ_max_ = 26.4°, θ_min_ = 3.5°
ω scans	*h* = −18→18
16399 measured reflections	*k* = −6→6
2788 independent reflections	*l* = −22→22

### Refinement

**Table:**

Refinement on *F*^2^	Secondary atom site location: difference Fourier map
Least-squares matrix: full	Hydrogen site location: inferred from neighbouring sites
*R*[*F*^2^ > 2σ(*F*^2^)] = 0.036	H atoms treated by a mixture of independent and constrained refinement
*wR*(*F*^2^) = 0.096	*w* = 1/[σ^2^(*F*_o_^2^) + (0.0525*P*)^2^ + 0.2968*P*] where *P* = (*F*_o_^2^ + 2*F*_c_^2^)/3
*S* = 1.03	(Δ/σ)_max_ < 0.001
2788 reflections	Δρ_max_ = 0.28 e Å^−^^3^
195 parameters	Δρ_min_ = −0.19 e Å^−^^3^
0 restraints	Extinction correction: SHELXL97 (Sheldrick, 2008), Fc^*^=kFc[1+0.001xFc^2^λ^3^/sin(2θ)]^-1/4^
Primary atom site location: structure-invariant direct methods	Extinction coefficient: 0.0097 (15)

### Special details

**Table:**

Geometry. All e.s.d.'s (except the e.s.d. in the dihedral angle between two l.s. planes) are estimated using the full covariance matrix. The cell e.s.d.'s are taken into account individually in the estimation of e.s.d.'s in distances, angles and torsion angles; correlations between e.s.d.'s in cell parameters are only used when they are defined by crystal symmetry. An approximate (isotropic) treatment of cell e.s.d.'s is used for estimating e.s.d.'s involving l.s. planes.
Refinement. Refinement of *F*^2^ against ALL reflections. The weighted *R*-factor *wR* and goodness of fit *S* are based on *F*^2^, conventional *R*-factors *R* are based on *F*, with *F* set to zero for negative *F*^2^. The threshold expression of *F*^2^ > σ(*F*^2^) is used only for calculating *R*-factors(gt) *etc*. and is not relevant to the choice of reflections for refinement. *R*-factors based on *F*^2^ are statistically about twice as large as those based on *F*, and *R*- factors based on ALL data will be even larger.

### Fractional atomic coordinates and isotropic or equivalent isotropic displacement parameters (Å^2^)

**Table:**

	*x*	*y*	*z*	*U*_iso_*/*U*_eq_	
N1	0.49215 (7)	0.2717 (2)	0.90078 (6)	0.0246 (2)	
H1	0.5157 (11)	0.129 (3)	0.9083 (9)	0.033 (4)*	
N2	−0.13564 (7)	0.9965 (2)	0.86242 (7)	0.0307 (3)	
O1	0.52173 (6)	0.69920 (16)	0.90359 (6)	0.0319 (2)	
O2	0.10753 (6)	0.21455 (17)	0.82463 (6)	0.0321 (2)	
O3	−0.12604 (8)	1.1019 (2)	0.92505 (7)	0.0495 (3)	
O4	−0.19763 (7)	1.0515 (2)	0.80818 (6)	0.0418 (3)	
C1	0.54954 (8)	0.4769 (2)	0.91080 (7)	0.0234 (3)	
C2	0.65154 (9)	0.4133 (2)	0.93362 (8)	0.0290 (3)	
H2A	0.6700	0.4115	0.9900	0.035*	
H2B	0.6620	0.2402	0.9149	0.035*	
C3	0.71154 (9)	0.6026 (3)	0.90187 (9)	0.0352 (3)	
H3A	0.7763	0.5540	0.9183	0.053*	
H3B	0.7023	0.7740	0.9209	0.053*	
H3C	0.6947	0.6019	0.8460	0.053*	
C11	0.39403 (8)	0.2759 (2)	0.88371 (7)	0.0226 (3)	
C12	0.34820 (9)	0.0773 (2)	0.91224 (8)	0.0291 (3)	
H12	0.3826	−0.0495	0.9437	0.035*	
C13	0.25238 (9)	0.0633 (2)	0.89496 (8)	0.0302 (3)	
H13	0.2215	−0.0714	0.9148	0.036*	
C14	0.20289 (8)	0.2483 (2)	0.84859 (7)	0.0250 (3)	
C15	0.24723 (9)	0.4488 (2)	0.82019 (7)	0.0270 (3)	
H15	0.2124	0.5757	0.7890	0.032*	
C16	0.34320 (9)	0.4629 (2)	0.83775 (7)	0.0258 (3)	
H16	0.3738	0.5994	0.8185	0.031*	
C21	0.05086 (8)	0.4152 (2)	0.83521 (7)	0.0251 (3)	
C22	−0.02514 (9)	0.4618 (3)	0.77680 (7)	0.0309 (3)	
H22	−0.0347	0.3634	0.7313	0.037*	
C23	−0.08678 (9)	0.6532 (3)	0.78568 (7)	0.0306 (3)	
H23	−0.1389	0.6875	0.7464	0.037*	
C24	−0.07090 (8)	0.7935 (2)	0.85284 (7)	0.0255 (3)	
C25	0.00430 (9)	0.7460 (3)	0.91150 (7)	0.0302 (3)	
H25	0.0134	0.8432	0.9572	0.036*	
C26	0.06596 (9)	0.5553 (3)	0.90256 (7)	0.0299 (3)	
H26	0.1179	0.5209	0.9420	0.036*	

### Atomic displacement parameters (Å^2^)

**Table:**

	*U*^11^	*U*^22^	*U*^33^	*U*^12^	*U*^13^	*U*^23^
N1	0.0236 (5)	0.0169 (5)	0.0340 (6)	0.0039 (4)	0.0074 (4)	0.0037 (4)
N2	0.0215 (5)	0.0311 (6)	0.0381 (6)	−0.0002 (4)	0.0029 (5)	−0.0022 (5)
O1	0.0261 (5)	0.0180 (4)	0.0503 (6)	0.0026 (4)	0.0045 (4)	0.0018 (4)
O2	0.0229 (5)	0.0270 (5)	0.0462 (6)	−0.0025 (4)	0.0065 (4)	−0.0104 (4)
O3	0.0393 (6)	0.0555 (7)	0.0490 (7)	0.0155 (5)	−0.0018 (5)	−0.0224 (6)
O4	0.0289 (5)	0.0452 (6)	0.0466 (6)	0.0089 (4)	−0.0032 (4)	0.0036 (5)
C1	0.0237 (6)	0.0209 (6)	0.0264 (6)	0.0024 (5)	0.0068 (5)	0.0025 (5)
C2	0.0255 (6)	0.0230 (6)	0.0387 (7)	0.0032 (5)	0.0070 (5)	0.0058 (5)
C3	0.0265 (7)	0.0322 (7)	0.0493 (8)	0.0022 (6)	0.0133 (6)	0.0065 (6)
C11	0.0240 (6)	0.0196 (5)	0.0253 (6)	0.0015 (4)	0.0078 (5)	−0.0020 (4)
C12	0.0291 (7)	0.0210 (6)	0.0382 (7)	0.0034 (5)	0.0090 (5)	0.0068 (5)
C13	0.0297 (7)	0.0218 (6)	0.0418 (7)	−0.0017 (5)	0.0136 (6)	0.0038 (5)
C14	0.0222 (6)	0.0234 (6)	0.0303 (6)	−0.0001 (5)	0.0073 (5)	−0.0063 (5)
C15	0.0269 (6)	0.0237 (6)	0.0292 (6)	0.0023 (5)	0.0030 (5)	0.0030 (5)
C16	0.0262 (6)	0.0231 (6)	0.0283 (6)	−0.0016 (5)	0.0062 (5)	0.0040 (5)
C21	0.0204 (6)	0.0243 (6)	0.0317 (7)	−0.0026 (5)	0.0082 (5)	−0.0015 (5)
C22	0.0286 (7)	0.0361 (7)	0.0269 (6)	−0.0046 (5)	0.0033 (5)	−0.0081 (5)
C23	0.0228 (6)	0.0382 (7)	0.0277 (7)	−0.0020 (5)	−0.0018 (5)	−0.0015 (5)
C24	0.0188 (6)	0.0273 (6)	0.0306 (6)	−0.0013 (5)	0.0055 (5)	−0.0002 (5)
C25	0.0260 (6)	0.0372 (7)	0.0260 (6)	0.0020 (5)	0.0018 (5)	−0.0077 (6)
C26	0.0231 (6)	0.0365 (7)	0.0275 (7)	0.0029 (5)	−0.0007 (5)	−0.0024 (5)

### Geometric parameters (Å, °)

**Table:**

N1—C1	1.3613 (16)	C12—C13	1.3954 (19)
N1—C11	1.4270 (16)	C12—H12	0.9500
N1—H1	0.825 (17)	C13—C14	1.3849 (18)
N2—O4	1.2273 (15)	C13—H13	0.9500
N2—O3	1.2314 (15)	C14—C15	1.3910 (18)
N2—C24	1.4678 (16)	C15—C16	1.3976 (18)
O1—C1	1.2339 (15)	C15—H15	0.9500
O2—C21	1.3842 (15)	C16—H16	0.9500
O2—C14	1.4047 (15)	C21—C26	1.3899 (18)
C1—C2	1.5233 (17)	C21—C22	1.3938 (18)
C2—C3	1.5201 (18)	C22—C23	1.389 (2)
C2—H2A	0.9900	C22—H22	0.9500
C2—H2B	0.9900	C23—C24	1.3875 (18)
C3—H3A	0.9800	C23—H23	0.9500
C3—H3B	0.9800	C24—C25	1.3894 (18)
C3—H3C	0.9800	C25—C26	1.3871 (18)
C11—C12	1.3965 (17)	C25—H25	0.9500
C11—C16	1.3972 (17)	C26—H26	0.9500
			
C1—N1—C11	126.93 (10)	C14—C13—H13	120.4
C1—N1—H1	117.5 (11)	C12—C13—H13	120.4
C11—N1—H1	115.4 (11)	C13—C14—C15	120.99 (11)
O4—N2—O3	122.86 (12)	C13—C14—O2	118.22 (11)
O4—N2—C24	118.75 (11)	C15—C14—O2	120.49 (11)
O3—N2—C24	118.38 (11)	C14—C15—C16	119.71 (11)
C21—O2—C14	117.81 (9)	C14—C15—H15	120.1
O1—C1—N1	123.01 (11)	C16—C15—H15	120.1
O1—C1—C2	121.87 (11)	C11—C16—C15	119.90 (11)
N1—C1—C2	115.10 (10)	C11—C16—H16	120.0
C3—C2—C1	112.62 (10)	C15—C16—H16	120.0
C3—C2—H2A	109.1	O2—C21—C26	121.72 (11)
C1—C2—H2A	109.1	O2—C21—C22	116.88 (11)
C3—C2—H2B	109.1	C26—C21—C22	121.31 (12)
C1—C2—H2B	109.1	C23—C22—C21	119.47 (12)
H2A—C2—H2B	107.8	C23—C22—H22	120.3
C2—C3—H3A	109.5	C21—C22—H22	120.3
C2—C3—H3B	109.5	C24—C23—C22	118.89 (12)
H3A—C3—H3B	109.5	C24—C23—H23	120.6
C2—C3—H3C	109.5	C22—C23—H23	120.6
H3A—C3—H3C	109.5	C23—C24—C25	121.81 (12)
H3B—C3—H3C	109.5	C23—C24—N2	119.11 (11)
C12—C11—C16	119.52 (11)	C25—C24—N2	119.08 (11)
C12—C11—N1	117.63 (11)	C26—C25—C24	119.30 (12)
C16—C11—N1	122.80 (11)	C26—C25—H25	120.4
C13—C12—C11	120.68 (12)	C24—C25—H25	120.4
C13—C12—H12	119.7	C25—C26—C21	119.22 (12)
C11—C12—H12	119.7	C25—C26—H26	120.4
C14—C13—C12	119.20 (11)	C21—C26—H26	120.4
			
C11—N1—C1—O1	−2.4 (2)	C14—C15—C16—C11	−0.06 (19)
C11—N1—C1—C2	176.22 (11)	C14—O2—C21—C26	−43.11 (17)
O1—C1—C2—C3	−34.15 (18)	C14—O2—C21—C22	140.29 (12)
N1—C1—C2—C3	147.24 (12)	O2—C21—C22—C23	177.11 (11)
C1—N1—C11—C12	−148.08 (13)	C26—C21—C22—C23	0.5 (2)
C1—N1—C11—C16	34.49 (19)	C21—C22—C23—C24	0.0 (2)
C16—C11—C12—C13	0.35 (19)	C22—C23—C24—C25	−0.6 (2)
N1—C11—C12—C13	−177.16 (12)	C22—C23—C24—N2	179.81 (12)
C11—C12—C13—C14	0.4 (2)	O4—N2—C24—C23	−6.26 (18)
C12—C13—C14—C15	−1.02 (19)	O3—N2—C24—C23	172.74 (13)
C12—C13—C14—O2	172.67 (11)	O4—N2—C24—C25	174.14 (12)
C21—O2—C14—C13	129.12 (12)	O3—N2—C24—C25	−6.86 (18)
C21—O2—C14—C15	−57.16 (16)	C23—C24—C25—C26	0.8 (2)
C13—C14—C15—C16	0.85 (19)	N2—C24—C25—C26	−179.65 (12)
O2—C14—C15—C16	−172.70 (11)	C24—C25—C26—C21	−0.3 (2)
C12—C11—C16—C15	−0.53 (18)	O2—C21—C26—C25	−176.78 (12)
N1—C11—C16—C15	176.85 (11)	C22—C21—C26—C25	−0.3 (2)

### Hydrogen-bond geometry (Å, °)

**Table:**

*D*—H···*A*	*D*—H	H···*A*	*D*···*A*	*D*—H···*A*
N1—H1···O1^i^	0.825 (17)	2.255 (17)	3.0306 (13)	156.7 (15)
C25—H25···O3^ii^	0.95	2.42	3.2082 (17)	140
C23—H23···O4^iii^	0.95	2.53	3.3400 (16)	144

Symmetry codes: (i) *x*, *y*−1, *z*; (ii) −*x*, −*y*+2, −*z*+2; (iii) −*x*−1/2, *y*−1/2, −*z*+3/2.

## References

[bb1] Akhter, Z., Nigar, A., Razzaq, M. Y. & Siddiqi, H. M. (2007). *J. Organomet. Chem.* **692**, 3542–3546.

[bb2] Allen, F. H., Kennard, O., Watson, D. G., Brammer, L., Orpen, A. G. & Taylor, R. (1995). *International Tables for Crystallography*, Vol. C, edited by A. J. C. Wilson, pp. 685–706. Dordrecht: Kluwer.

[bb3] Bahadur, B. (1992). *Liquid Crystals: Applications and Uses*, Vol. 2. Singapore: World Scientific.

[bb4] Cârlescu, I., Hurduc, N., Scutaru, D., Câtânescu, O. & Chien, L. (2005). *Mol. Cryst. Liq. Cryst.* **439**, 1973-1989.

[bb5] Collings, P. J. (1990). In *Liquid Crystals: Nature’s Delicate Phase of Matter* Bristol: Adam Hilger.

[bb6] Collings, P. J. & Hird, M. (1997). *Introduction to Liquid Crystals* London: Taylor & Francis.

[bb7] Sheldrick, G. M. (2008). *Acta Cryst.* A**64**, 112–122. 10.1107/S0108767307043930 18156677

[bb8] Stoe & Cie (2001). *X-AREA* and *X-RED* Stoe & Cie, Darmstadt, Germany.

